# Pharmaceutical Compounding as a Pillar of Personalized Oncology: Current Applications, Emerging Technologies, and Future Perspectives

**DOI:** 10.3390/ph19071077

**Published:** 2026-07-13

**Authors:** Filipa Mascarenhas-Melo, Rafael Pinheiro, Francis Victor, Maria Eugénia Pina, Ana Figueiras

**Affiliations:** 1 Higher School of Health, Polytechnic Institute of Guarda, Rua Da Cadeia, 6300-307 Guarda, Portugal; filipamelo@ff.uc.pt; 2 BRIDGES—Biotechnology Research, Innovation and Design of Health Products, Polytechnic of Guarda, 6300-559 Guarda, Portugal; 3 Laboratory of Drug Development and Technologies, Faculty of Pharmacy, University of Coimbra, 3000-548 Coimbra, Portugal; rafaelferreirapinheiro@hotmail.com; 4 Department of Pharmacy, University Chenab, Gujarat 50700, Punjab, Pakistan; vfrancis030@gmail.com; 5 Department of Chemical Engineering, CERES—Chemical Engineering and Renewable Resources for Sustainability, University of Coimbra, 3030-790 Coimbra, Portugal; 6 Faculty of Pharmacy, Research Institute for Medicine (iMed.ULisboa), University of Lisbon, 1649-004 Lisbon, Portugal; 7 REQUIMTE/LAQV, Group of Pharmaceutical Technology, Faculty of Pharmacy, University of Coimbra, 3000-548 Coimbra, Portugal

**Keywords:** pharmaceutical compounding, oncology pharmacy, personalized oncology, precision medicine, individualized therapies, advanced pharmaceutical technologies

## Abstract

Personalized oncology is transforming cancer care by tailoring therapeutic strategies to the molecular and clinical characteristics of individual patients. However, increasing treatment complexity, interpatient variability, and the growing use of advanced therapeutics challenge the limitations of standardized medicines. This review examines pharmaceutical compounding as a fundamental component enabling the delivery of individualized oncology treatments. A literature search was conducted in PubMed/MEDLINE, Scopus, and Web of Science, using a predefined search strategy detailed in the manuscript. This narrative review of the literature was conducted to evaluate the application of pharmaceutical compounding in modern oncology practice. The analysis includes immunotherapy, nanotechnology-based drug delivery systems, genomic-guided therapy, and combination treatment strategies. Emerging technologies, such as artificial intelligence, three-dimensional printing, and robotic compounding, were also assessed, alongside regulatory frameworks, safety challenges, and quality considerations. The main findings of this study show that compounded medications support individualized care through dose adjustment, modification of dosage forms, and exclusion of unsuitable excipients, particularly in pediatric oncology, rare cancers, and patients with specific needs. The magistral and officinal preparations help maintain continuity of care when commercial formulations are unavailable. In addition, technological advances are improving the precision, reproducibility, and safety of compounding processes, and pharmacists are centrally involved in the design, preparation, quality assurance, and regulatory oversight of these therapies. In conclusion, pharmaceutical compounding remains an essential component of personalized oncology, enabling patient-centered and adaptable treatment strategies. The expanding engagement of pharmacists, together with advances in technology and evolving regulatory frameworks, is essential to ensuring the safe and effective implementation of individualized therapies in oncology care.

## 1. Introduction

Oncology is the branch of medicine concerned with the study, diagnosis, and management of cancer, a heterogeneous group of diseases characterized by uncontrolled cellular proliferation with the potential for invasion and metastasis. Cancer remains a major global health challenge, with the most commonly diagnosed malignancies including breast, lung, colorectal, and prostate cancers. Despite advances in prevention and treatment, cancer-related mortality remains high, reflecting differences in tumor biology, stage at diagnosis, and access to care [1,2,3,4].

The global cancer burden continues to rise substantially. Recent estimates from GLOBOCAN project that the number of new cancer cases will exceed 35 million annually by 2050, driven largely by population aging, growth, and changes in exposure to modifiable risk factors such as tobacco use, alcohol consumption, obesity, and environmental pollutants [3,5,6]. This increasing burden places significant pressure on healthcare systems and highlights the need for more effective and individualized therapeutic strategies.

Cancer management is inherently complex and typically involves a multidisciplinary approach, including surgery, chemotherapy, radiotherapy, targeted therapy, and immunotherapy. Although these modalities have significantly improved patient outcomes, they are not universally suitable for all patients due to variability in clinical characteristics, treatment tolerability, and tumor heterogeneity, which contribute to therapeutic resistance and variable responses [7,8,9]. As a result, there is an increasing need for therapeutic approaches that can be tailored to individual patient requirements.

In this context, advances in precision oncology have enabled the molecular stratification of tumors and the development of targeted therapeutic strategies guided by tumor-specific biological characteristics [9,10]. However, the complexity and diversity of cancer biology continue to present challenges in achieving optimal individualized treatment outcomes.

Within this framework, pharmaceutical compounding constitutes a key element in enabling the customization of oncology treatments. Compounded medications allow for dose adjustments, alternative dosage forms, and the exclusion of problematic excipients, which is particularly relevant in pediatric oncology, rare cancers, and patients with specific clinical needs. Pharmacists are central to this process, contributing to the design, preparation, and safe use of individualized therapies and facilitating the practical implementation of personalized medicine in clinical settings [11,12,13].

This study examines recent advances in compounded oncology medications and highlights the evolving contributions of pharmacists to the delivery of safe, effective, and patient-centered cancer care, with particular emphasis on emerging technologies and future directions in personalized medicine.

## 2. Search Strategy

A literature search was conducted in PubMed/MEDLINE, Scopus, and Web of Science to identify relevant studies on pharmaceutical compounding in oncology. The search included articles published in English from 2000 to June 2026.

Searches were performed using combinations of keywords related to pharmaceutical compounding and oncology, including “personalized medicine,” “immunotherapy,” “nanotechnology,” and “genomic-guided therapy,” combined using Boolean operators (AND, OR).

Studies were included if they addressed pharmaceutical compounding or individualized oncology therapies, or relevant enabling technologies. Reviews and original research articles were considered. Non-oncology studies, non-relevant publications, and non-peer-reviewed sources were excluded. Additional relevant articles were identified through manual screening of reference lists.

## 3. Global Cancer Burden and Epidemiology

**Figure 1:**
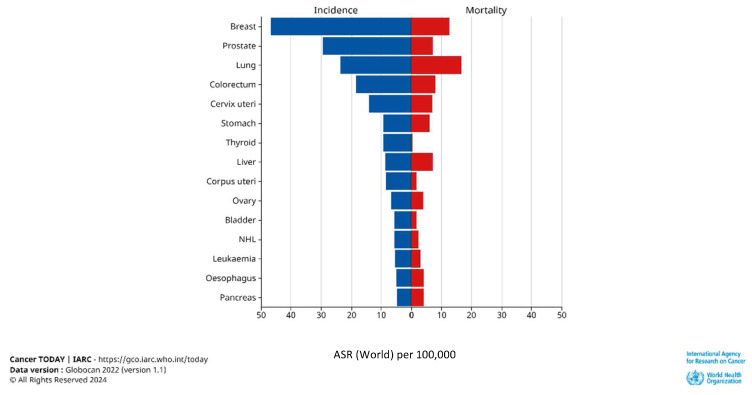
Age-standardized rates (ASR, per 100,000 population) of the most common cancers worldwide for incidence (blue) and mortality (red), based on GLOBOCAN 2022 estimates of cancer incidence and mortality worldwide. Adapted from [6].

According to the International Agency for Research on Cancer (IARC), the most common cancers worldwide are breast cancer (affecting about 46.8 per 100,000 individuals), prostate cancer (29.4), lung cancer (23.6), and colorectal cancer (18.4) [1]. In contrast, cancer mortality rates remain highest for lung (16.8), breast (12.7), and colorectal (8.1) cancers (Figure 1) [6]. The divergence between incidence and mortality reflects differences in disease aggressiveness, stage at diagnosis, and access to effective prevention, early detection, and treatment across regions. These global cancer statistics underscore the ongoing need to strengthen cancer control strategies, including risk factor mitigation, improved screening uptake, and equitable access to high-quality care.

In light of this increasing burden, a comprehensive understanding of cancer etiology is essential and is addressed in the following section.

## 4. Etiology and Molecular Pathogenesis of Cancer

Cancer risk factors are broadly classified into intrinsic (non-modifiable) and non-intrinsic (partially or fully modifiable) determinants. Intrinsic factors are primarily driven by the basal mutation rate inherent to all dividing cells and arise from stochastic errors during DNA replication, as well as inherited genetic susceptibility. In contrast, non-intrinsic factors encompass a heterogeneous range of endogenous biological processes and exogenous exposures that contribute to carcinogenesis and are, to varying degrees, preventable or modifiable. Representative examples of these risk factors are summarized in Table 1 [2]. Epidemiological evidence indicates that non-intrinsic factors account for a substantial proportion of cancer risk at the population level, reinforcing the importance of prevention strategies [2].

Cancer etiology involves a complex interplay of genetic mutations and environmental influences that drive the multistep process of carcinogenesis. DNA damage arises from both endogenous and exogenous sources, ultimately compromising genomic integrity and promoting malignant transformation.

Endogenous DNA damage results from normal cellular processes, including oxidative stress, spontaneous hydrolytic reactions, and aberrant DNA methylation. In particular, reactive oxygen species generated through biochemical reactions such as the Fenton reaction induce DNA base modifications and strand breaks, while epigenetic alterations may interfere with transcriptional regulation and DNA repair mechanisms.

Exogenous sources of DNA damage include ionizing and non-ionizing radiation, chemical carcinogens, and environmental pollutants. Alkylating agents present in tobacco smoke, industrial emissions, and air pollution induce DNA adduct formation and mutations that, if not adequately repaired, contribute to carcinogenesis [14]. Persistent exposure to these agents increases mutational burden and accelerates malignant transformation.

At the molecular level, cancer is characterized by genomic instability, defined as the accumulation of genetic alterations affecting genes involved in cell proliferation, survival, and genome maintenance [15]. This instability is driven by key alterations in oncogenes (e.g., KRAS, HER2), which promote uncontrolled proliferation when activated, and tumor suppressor genes (e.g., TP53, BRCA1/2), which normally regulate cell-cycle arrest and apoptosis but contribute to tumorigenesis when inactivated [15,16]. These molecular alterations disrupt essential cellular processes, including DNA damage response, cell-cycle checkpoint control, apoptosis, and growth-regulatory signaling pathways. As a consequence, cells progressively acquire a selective growth advantage, leading to malignant transformation. Collectively, cancer development arises from the interaction between unavoidable intrinsic mechanisms and modifiable environmental and lifestyle exposures. This duality not only underpins carcinogenesis but also highlights opportunities for prevention and provides the biological basis for precision oncology and targeted therapeutic strategies [2].

## 5. Personalization of Cancer Therapy

Customization of therapy refers to the process by which pharmacological treatment is tailored to the individual patient based on clinical condition, disease characteristics, molecular profile, and patient-specific factors, with the aim of optimizing efficacy and minimizing toxicity.

Cancer management requires a multidisciplinary approach involving surgery, radiotherapy, and systemic therapies, including chemotherapy, hormone therapy, targeted therapy, and immunotherapy. Treatment selection is determined by tumor type, stage, molecular characteristics, and the patient’s overall clinical status [3]. Within systemic therapy, chemotherapy may be administered in neoadjuvant, adjuvant, or palliative settings depending on therapeutic intent. Neoadjuvant chemotherapy is used to reduce tumor burden and facilitate surgical resection, while adjuvant chemotherapy aims to eradicate residual microscopic disease and reduce recurrence risk. In advanced disease, palliative chemotherapy is primarily directed toward symptom control, improvement of quality of life, and potential survival prolongation, while minimizing treatment-related toxicity [6,7].

In parallel, advances in precision oncology and immunotherapy have significantly expanded treatment individualization. Molecularly targeted therapies, such as EGFR, ALK, and HER2 inhibitors, are selected based on specific tumor alterations, while immune checkpoint inhibitors enhance anti-tumor immune responses in selected patient populations. These developments have increased the proportion of patients eligible for personalized treatment strategies and have reshaped contemporary oncological practice [7,10,11].

Despite these advances, therapeutic decision-making remains complex due to interpatient variability, tumor heterogeneity, and differences in drug response. This growing complexity highlights the need for individualized approaches to pharmacotherapy and provides the clinical foundation for the development of personalized treatment strategies, including pharmaceutical compounding.

## 6. Cancer Biology and Molecular Pathogenesis

Cancer development is driven by the progressive accumulation of genetic and epigenetic alterations that disrupt normal cellular homeostasis and promote malignant transformation. DNA damage arises from both endogenous and exogenous sources. Endogenous processes, including oxidative stress generated through Fenton-type reactions, can induce DNA base modifications and strand breaks, while aberrant DNA methylation may interfere with transcriptional regulation and DNA repair mechanisms.

Exogenous sources of DNA damage include ionizing and non-ionizing radiation, chemical carcinogens, and environmental pollutants. Alkylating agents present in tobacco smoke, industrial exposures, and air pollution induce DNA adduct formation and mutations that, if not adequately repaired, contribute to carcinogenesis [14]. Persistent exposure to these agents increases mutational burden and accelerates malignant transformation. These molecular alterations disrupt essential cellular processes, including cell-cycle checkpoint control, DNA damage response, and repair pathways.

**Figure 2:**
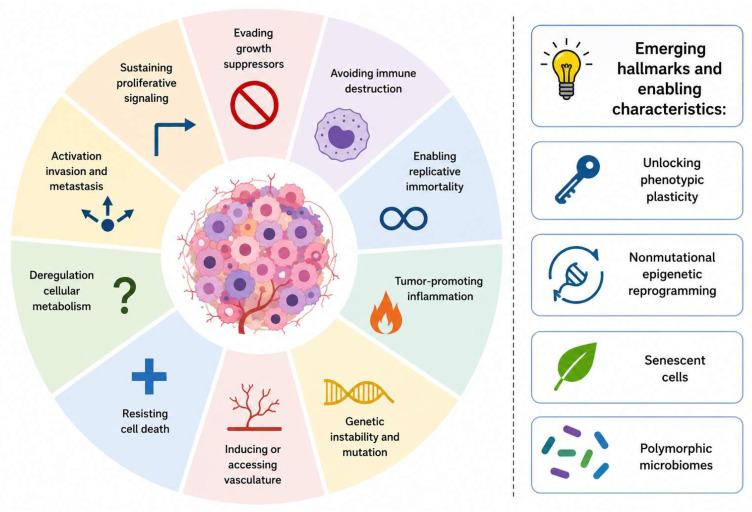
Representation of the hallmarks of cancer (2022 update). The left panel illustrates the eight core functional capabilities and two enabling characteristics that underpin tumor development and progression. The right panel incorporates emerging hallmarks, including phenotypic plasticity and cellular senescence, together with enabling characteristics such as non-mutational epigenetic reprogramming and polymorphic microbiomes.

As a consequence, cancer cells acquire hallmark capabilities such as sustained proliferative signaling, resistance to apoptosis, replicative immortality, angiogenesis, and the ability to invade and metastasize [4]. The tumor microenvironment, including inflammatory cells and angiogenic mediators, further supports tumor progression and survival [17]. The hallmarks of cancer are summarized in Figure 2.

Notably, while some oncogenic mutations arise spontaneously or are inherited, a substantial proportion of cancer risk is associated with non-intrinsic factors. Lifestyle and environmental exposures such as tobacco smoke, ultraviolet radiation, alcohol consumption, diet, oncogenic viral infections (e.g., human papillomavirus and hepatitis B and C viruses), and occupational carcinogens contribute significantly to mutational burden. A 2018 analysis by Wu et al. reinforced that non-intrinsic factors are the dominant contributors to overall cancer risk at the population level [2]. This etiological heterogeneity provides a strong rationale for personalized oncology, as optimal treatment strategies often require tailoring to the molecular characteristics of each patient’s tumor [18]. Tumors arising in different patients may harbor distinct molecular alterations that influence therapeutic response. For example, a patient whose lung tumor harbors an EGFR mutation will benefit from EGFR tyrosine kinase inhibitors, whereas another patient with the same clinical diagnosis but without the mutation would not [18]. This highlights the importance of molecular profiling in guiding individualized treatment strategies.

Therapeutically, the hallmarks of cancer represent critical targets for intervention. Conventional cytotoxic chemotherapies act non-specifically on rapidly dividing cells, primarily exploiting sustained proliferative signaling, but are often associated with significant toxicity to normal tissues. Advances in cancer biology have led to the identification of additional emerging hallmarks, including phenotypic plasticity, non-mutational epigenetic reprogramming, modulation by polymorphic microbiomes, and the accumulation of senescent cells. These features further expand the functional capabilities that cancer cells can acquire to sustain tumor growth and progression [4].

## 7. Innovative Advances in Cancer Therapies

Newer targeted therapies and immunotherapies are designed to more precisely target cancer-specific abnormalities. For instance, angiogenesis inhibitors disrupt tumor neovascularization signaling. Neovascularization is triggered when tissues require oxygen and nutrients and is regulated by a balance between pro-angiogenic and anti-angiogenic mediators. Major pro-angiogenic factors include vascular endothelial growth factor (VEGF), basic fibroblast growth factor (bFGF), tumor necrosis factor-α (TNF-α), and nitric oxide (NO). Conversely, angiogenesis is inhibited by factors such as interleukin-12 (IL-12), thrombospondin-1 (TSP-1), tissue inhibitors of metalloproteinases (TIMPs), angiotensin, and interferon-α. Immune checkpoint inhibitors restore immune recognition of cancer cells [4,19,20]. However, tumors are highly adaptable; redundant signaling pathways and intratumoral genetic heterogeneity contribute to the development of drug resistance over time [21]. Consequently, combination therapies and sequential treatment strategies are often required to address the multifaceted nature of the disease [22].

Recent advances in oncology therapeutics reflect a broader shift toward personalized medicine, in which treatment strategies are increasingly guided by molecular tumor characteristics and patient-specific factors. This includes the integration of targeted agents, immunotherapeutic approaches, and rational combination regimens designed to improve efficacy while minimizing toxicity.

### 7.1. Immunotherapy Advancements: Monoclonal Antibodies and Checkpoint Inhibitors

Immunotherapy has emerged as a transformative approach in cancer treatment, leveraging the patient’s own immune system to identify and eliminate tumor cells. Among the most impactful strategies are monoclonal antibodies (mAbs) and immune checkpoint inhibitors (ICIs). Monoclonal antibodies are laboratory-engineered proteins designed to bind specific antigens on cancer cells. By targeting unique markers (such as CD20 on B-cell lymphomas or HER2 on breast cancer cells), mAbs can directly flag those cells for immune attack or block growth signals [1]. Some mAbs also serve as delivery vehicles in antibody-drug conjugates, ferrying potent cytotoxic agents directly to cancer cells. Immune checkpoint inhibitors, on the other hand, work by releasing the “brakes” on T cells. Drugs like pembrolizumab and nivolumab (anti-PD-1 antibodies), atezolizumab (anti-PD-L1), and ipilimumab (anti-CTLA-4) block inhibitory signals that tumors use to deactivate T cells. By inhibiting these checkpoints, the patient’s T cells can better recognize and kill cancer cells. Checkpoint inhibitors have revolutionized the treatment of melanoma, lung cancer, renal cell carcinoma, and many other malignancies, leading to durable remissions in a subset of patients who previously had few options [2]. Recent clinical trials have expanded their use beyond the initial indications; for example, immune checkpoint inhibitors (ICIs) are now being used in microsatellite instability-high colorectal cancers, certain gastric cancers, and even in earlier-stage disease as neoadjuvant therapy. Despite these successes, challenges remain: not all patients respond (or they eventually develop resistance), and immune-related adverse effects (irAEs) such as colitis, dermatitis, and endocrinopathies can be severe. Ongoing research is exploring biomarkers to predict who will benefit from immunotherapy and how to mitigate toxicity [3].

### 7.2. Emerging Immunotherapies

**Figure 3:**
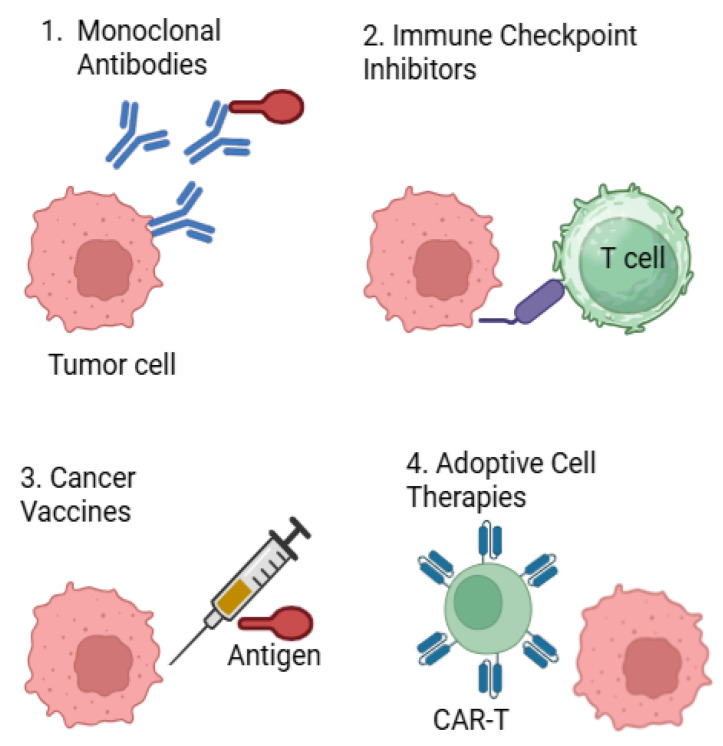
Contemporary cancer immunotherapies encompass several approaches: (1) Monoclonal antibodies (mAbs), synthetic proteins targeting tumor-associated antigens (e.g., HER2, CD20) or immune checkpoints; (2) Immune checkpoint inhibitors (ICIs), a subclass of mAbs that block inhibitory receptors such as PD-1/PD-L1 and CTLA-4, thereby enhancing T-cell-mediated anti-tumor responses; (3) Cancer vaccines, including mRNA-, peptide-, and dendritic cell-based platforms, which stimulate immune recognition of tumor antigens; and (4) Adoptive cell therapies, such as CAR T-cell therapy, in which patient-derived T cells are genetically engineered to express chimeric antigen receptors targeting tumor cells and reinfused to mediate tumor killing. These approaches are increasingly used alone or in combination and have substantially expanded therapeutic options in oncology.

In addition to mAbs and ICIs, newer forms of immunotherapy are gaining traction. Adoptive cell therapies—most notably CAR T-cell therapy—involve genetically engineering a patient’s own immune cells to better target cancer. Chimeric Antigen Receptor (CAR) T-cells have shown remarkable efficacy in refractory leukemias and lymphomas, achieving high remission rates by targeting antigens like CD19 on B cells. These “living drugs” exemplify personalized therapy, as each batch of CAR-T cells is uniquely made for the patient from whom they were derived [23]. Other immunotherapeutic approaches include therapeutic cancer vaccines (e.g., sipuleucel-T for prostate cancer) and oncolytic viruses. Moreover, bi-specific T cell engagers (BiTEs) are antibody constructs that can bind a T-cell on one side and a tumor cell on the other, bringing them into proximity to induce cancer cell killing. Figure 3 illustrates the spectrum of current immunotherapy modalities—from monoclonal antibodies and checkpoint blockade to cancer vaccines and CAR T-cells [7]. Combination immunotherapy (using two ICIs together, or ICI plus another modality) is also being investigated to improve response rates. For instance, dual checkpoint blockade with anti-CTLA-4 and anti-PD-1 has become a standard of care in advanced melanoma, albeit with increased toxicity [2]. As immunotherapy techniques diversify, pharmacists are increasingly involved in managing their administration, for example, reconstituting and diluting mAbs for infusion, or handling CAR-T cell products, which require careful storage and thawing protocols.

The involvement of the pharmacist in compounding is less focused on traditional manipulation of raw active pharmaceutical ingredients and more centered on the preparation of complex sterile admixtures, including accurate dose calculations, aseptic reconstitution and dilution of biologics, and verification of vehicle compatibility and physicochemical stability. This is particularly relevant for monoclonal antibodies, which often require strict handling under cleanroom conditions and may exhibit limited stability following dilution or reconstitution.

The advent of immunotherapy has significantly improved outcomes in several cancers and even resulted in cures in diseases like metastatic melanoma that were once almost uniformly fatal. The need to personalize immunotherapy is evident in how patient selection is performed: tumors are now often tested for PD-L1 expression, microsatellite instability, or specific immune gene signatures to predict ICI benefit. For pharmacists, immunotherapy introduces new considerations in compounding and dispensing; for example, many mAbs require gentle handling (they are proteins that can denature with shaking or improper temperature), and dosing might be weight-based or fixed depending on the drug, requiring careful calculation and preparation. Furthermore, managing immune-related toxicities can involve high-dose corticosteroids or other immunosuppressants, which pharmacists help to dose-adjust and compound (e.g., topical steroid formulations for certain dermatologic irAEs) [10].

Immunotherapy, as a cornerstone of personalized medicine, exemplifies the transition of oncology away from standardized treatment paradigms toward more individualized therapeutic approaches, while requiring coordinated multidisciplinary management in which pharmacists contribute substantially to the safe and effective implementation of these therapies in clinical practice [15].

### 7.3. Lipid–Polymer Hybrid Nanoparticles (Lphnps): Targeted Drug Delivery

Nanotechnology has enabled the development of advanced drug delivery systems aimed at improving the precision and therapeutic index of anticancer agents. Among these, lipid–polymer hybrid nanoparticles (LPHNPs) represent a promising platform that combines the structural stability of polymeric nanoparticles with the biocompatibility of lipid-based carriers. Typically, LPHNPs consist of a biodegradable polymeric core encapsulating the therapeutic payload, surrounded by a lipid shell that enhances stability and facilitates surface functionalization with targeting ligands. This architecture enables controlled drug release and improved tumor-selective delivery, thereby reducing systemic toxicity [24].

Preclinical studies have demonstrated the potential of LPHNPs to enhance intratumoral drug accumulation and overcome mechanisms of multidrug resistance. For example, Yang et al. reported improved delivery of chemotherapeutic agents and nucleic acids using LPHNP systems in tumor models, with enhanced efficacy compared with conventional formulations [25]. Targeting strategies, including antibody- or peptide-mediated surface modification, enable selective binding to tumor-associated receptors such as HER2 or folate receptors. In addition, LPHNPs exploit the enhanced permeability and retention (EPR) effect, allowing preferential accumulation within tumor tissue, followed by controlled or stimuli-responsive drug release triggered by acidic pH or enzymatic activity in the tumor microenvironment. Some formulations further enable co-delivery of multiple agents, supporting combination therapy or simultaneous drug–gene strategies to counteract resistance mechanisms. From a pharmaceutical practice perspective, lipid–polymer hybrid nanoparticles (LPHNPs) introduce specific considerations in handling, preparation, and administration that extend beyond classical compounding of the nanocarrier itself. During reconstitution, dilution, and transfer steps, these nanomedicines may be exposed to physicochemical stresses that can compromise particle integrity, including shear stress, interfacial stress, and adsorption to container or tubing surfaces, potentially leading to lipid membrane disruption, drug leakage, or changes in particle size distribution. In addition, filtration and agitation steps must be carefully controlled to avoid destabilization of the hybrid architecture or unintended aggregation. Many nanoparticle-based anticancer agents, including liposomal formulations, therefore require validated handling protocols to preserve colloidal stability prior to administration. Pharmacists play a key role in ensuring the integrity of preparation procedures through appropriate selection of diluents, avoidance of incompatible filtration systems, and minimization of mechanical stress during manipulation and transfer steps.

Pharmacists contribute substantially to ensuring the integrity of preparation procedures through the appropriate selection of diluents, avoidance of incompatible filtration systems, and strict adherence to manufacturer-specific handling recommendations [26]. As nanomedicines increasingly enter clinical trials and routine oncology practice, pharmacists may also be involved in clinical trial preparation, quality assurance, and safe dispensing of investigational formulations.

Emerging research is also exploring externally triggered nanoparticle systems, including photothermal and ultrasound-responsive platforms, designed to enable spatially controlled drug release at tumor sites [27,28]. Although still largely experimental, these technologies illustrate the ongoing evolution of precision drug delivery systems.

Overall, LPHNPs represent an important convergence of nanotechnology and oncology pharmacotherapy, offering improved tumor targeting and reduced off-target toxicity. Their continued development is closely aligned with the principles of precision medicine and further underscores the growing contribution of pharmacists to the safe integration and clinical implementation of advanced therapeutic technologies.

As shown in Table 2, the range of therapeutic agents investigated in lipid–polymer hybrid nanoparticles (LPHNPs) has expanded substantially, although most studies remain concentrated in the oncology field. Anticancer drugs continue to represent the largest category of encapsulated cargoes, reflecting both the need to improve tumor-specific drug accumulation and the ability of LPHNPs to reduce the systemic toxicity associated with many chemotherapeutic agents. However, the literature also demonstrates a growing interest in the delivery of nucleic acids, including siRNA, miRNA, mRNA and plasmid DNA, as well as proteins, peptides, vaccines and immunomodulatory molecules. This trend mirrors the broader shift in nanomedicine towards advanced therapeutics that require efficient intracellular delivery and protection from biological degradation.

The diversity of therapeutic agents reported to date highlights one of the principal advantages of LPHNPs: the possibility of combining the structural stability and controlled-release properties of polymeric systems with the biocompatibility and membrane-interacting characteristics of lipid-based carriers. As a result, these hybrid systems have been investigated not only to improve drug solubility and pharmacokinetics but also to enhance cellular uptake, protect sensitive biomolecules and enable targeted delivery. Nevertheless, despite the large number of promising preclinical studies, clinical translation remains limited. Many of the challenges identified in the broader nanomedicine field are also evident for LPHNPs, including manufacturing scalability, formulation reproducibility, long-term stability and the limited predictive value of conventional preclinical models. These issues continue to represent important barriers to the successful clinical implementation of LPHNP-based therapies [29,30].

**Table 2:** Therapeutic agents delivered via lipid–polymer hybrid nanoparticles (LPHNPs) by disease/application.

Disease/Application Area	Therapeutic Agents	Therapeutic Class	Delivery Purpose/Rationale	Key Application Notes	References
**Breast cancer**	Doxorubicin, Paclitaxel, Docetaxel (alone or in combination regimens)	Anthracycline antibiotic (doxorubicin); taxane chemotherapeutics (paclitaxel, docetaxel)	Enhance passive and active tumor targeting via EPR effect and surface functionalization, improve drug solubility and pharmacokinetic profiles, enable controlled and sustained release, and reduce dose-limiting systemic toxicity associated with anthracyclines and taxanes	One of the most extensively studied models for LPHNP-based drug delivery, particularly in breast cancer; widely investigated in both single-drug and combination chemotherapy strategies, including sequential and co-delivery systems, with extensive preclinical evidence demonstrating improved tumor accumulation and therapeutic efficacy	[31,32,33]
**Lung cancer**	Cisplatin, Paclitaxel, Gefitinib (alone or in combination regimens)	Platinum-based chemotherapeutic (cisplatin); taxane chemotherapeutic (paclitaxel); tyrosine kinase inhibitor (EGFR-targeted, gefitinib)	Overcome acquired and intrinsic drug resistance mechanisms in non-small cell lung cancer (NSCLC), enhance pulmonary and tumor-specific drug delivery via inhalable or systemic nanoparticle platforms, improve pharmacokinetic profiles, and reduce systemic toxicity associated with platinum and taxane-based chemotherapy	Widely investigated in nanoparticle-based NSCLC models, including LPHNP systems, to enhance tumor accumulation, improve intracellular drug delivery, and overcome multidrug resistance (MDR); combination chemotherapy and targeted therapy strategies (including EGFR inhibition) are frequently explored to achieve synergistic antitumor effects	[34,35,36]
**Colorectal cancer**	5-Fluorouracil; oxaliplatin; curcumin (alone or combination regimens)	Antimetabolite (5-FU); platinum-based chemotherapeutic (oxaliplatin); phytochemical/pleiotropic anticancer agent (curcumin)	Enhance systemic exposure; improve oral/colon-specific bioavailability; enable controlled and sustained release; reduce systemic toxicity associated with combination chemotherapy	Widely explored in colon-targeted and oral nanoparticulate systems, particularly in FOLFOX-based combination strategies; sustained-release formulations have been investigated to improve drug stability, reduce dosing frequency, and enhance therapeutic index in colorectal cancer models	[37,38,39,40]
**Prostate cancer**	Docetaxel; siRNA (androgen receptor-targeting)	Taxane chemotherapeutic (docetaxel); RNA interference therapeutic (gene silencing of androgen receptor signaling)	Enhance therapeutic efficacy through combined chemotherapy and androgen receptor gene silencing, overcome androgen receptor-driven resistance in castration-resistant prostate cancer (CRPC), and improve tumor-selective intracellular delivery while reducing systemic toxicity	Extensively investigated in CRPC nanomedicine as a co-delivery strategy integrating chemotherapy and gene silencing; PSMA-targeted nanoparticle systems (including lipid–polymer hybrid platforms) have demonstrated improved tumor accumulation, prolonged circulation time, and enhanced suppression of androgen receptor-mediated signaling pathways	[41,42,43]
**Glioblastoma/brain tumors**	Temozolomide; doxorubicin; siRNA; mRNA (alone or in combination strategies)	Alkylating agent (temozolomide); anthracycline chemotherapeutic (doxorubicin); RNA interference therapeutics (siRNA); mRNA-based gene therapeutics	Overcome the blood–brain barrier (BBB), enhance central nervous system (CNS) drug delivery, enable multi-modal therapy combining chemotherapy and gene regulation, and improve intracellular delivery of nucleic acids while reducing systemic toxicity	Widely investigated in glioblastoma nanomedicine using lipid–polymer hybrid and surface-functionalized nanoparticles designed for receptor-mediated BBB penetration; most studies focus on either chemotherapeutic delivery (e.g., temozolomide or doxorubicin) or nucleic acid delivery (siRNA/mRNA), with emerging interest in multifunctional platforms enabling co-delivery and enhanced tumor accumulation in brain tissues	[44,45,46]
**Pancreatic cancer**	Gemcitabine (alone or in combination regimens such as nab-paclitaxel or FOLFIRINOX components)	Nucleoside analog (gemcitabine); combination cytotoxic chemotherapy regimens	Enhance therapeutic efficacy by overcoming rapid metabolic degradation and intrinsic chemoresistance in pancreatic ductal adenocarcinoma (PDAC), improve tumor accumulation in a desmoplastic tumor microenvironment, and enable sustained drug exposure while reducing systemic toxicity	Gemcitabine represents the standard first-line chemotherapeutic agent for pancreatic cancer and is widely used in combination regimens (e.g., nab-paclitaxel or FOLFIRINOX). Extensive research has focused on nanoparticle-based delivery systems, including lipid, polymeric, and hybrid platforms, to improve pharmacokinetics, overcome stromal barriers, and enhance clinical efficacy	[47,48,49,50]
**Gene therapy (oncology)**	siRNA; miRNA; plasmid DNA; mRNA; CRISPR/Cas9 (gene editing systems, alone or in distinct delivery platforms)	RNA interference therapeutics (siRNA, miRNA); messenger RNA therapeutics; plasmid DNA gene therapy; genome editing systems (CRISPR/Cas9)	Enhance stability and protection of nucleic acid cargo against enzymatic degradation, improve cellular uptake and intracellular trafficking, enable efficient endosomal escape and nuclear delivery (for DNA and CRISPR systems), and overcome biological barriers limiting gene therapy efficacy in solid tumors	Major emerging area in oncology nanomedicine involving non-viral delivery systems, particularly lipid–polymer hybrid nanoparticles, lipid nanoparticles, and surface-functionalized nanocarriers. Current research focuses on improving delivery efficiency, tissue targeting, and safety profiles of diverse nucleic acid therapeutics, with increasing emphasis on CRISPR/Cas9 and mRNA-based approaches alongside RNA interference and plasmid DNA gene therapy	[51,52,53,54]
**Cancer immunotherapy**	mRNA vaccines; immune-modulating RNA constructs (alone or in combination with immunotherapy strategies)	mRNA-based cancer vaccines; immunomodulatory RNA therapeutics	Induce tumor-specific immune responses through antigen presentation and immune system activation, enhance stability and delivery of mRNA constructs using lipid-based nanocarriers, and enable modulation of innate and adaptive immune pathways for anti-tumor immunity	Rapidly expanding field of cancer immunotherapy strongly enabled by lipid nanoparticle (LNP) delivery platforms originally optimized during the COVID-19 pandemic. Current research focuses on personalized cancer vaccines, immune activation strategies, and combination therapies with immune checkpoint inhibitors, although most applications remain in early clinical or preclinical stages	[55,56,57,58]
**Antimicrobial therapy**	Vancomycin; ciprofloxacin (alone or in nanoparticle-based antimicrobial delivery systems)	Glycopeptide antibiotic (vancomycin); fluoroquinolone antibiotic (ciprofloxacin)	Enhance antimicrobial efficacy against biofilm-associated and multidrug-resistant infections by improving antibiotic penetration into the extracellular polymeric substance (EPS), increasing local drug concentration, and overcoming diffusion barriers and resistance mechanisms	Widely studied in lipid, polymeric, and lipid–polymer hybrid nanoparticle systems for anti-biofilm applications. Preclinical studies demonstrate enhanced penetration into bacterial biofilms, improved bactericidal activity compared to free antibiotics, and reduced systemic toxicity. These systems are primarily evaluated in in vitro and in vivo infection models	[59,60,61,62]
**Inflammatory diseases**	Dexamethasone; curcumin; NSAIDs (e.g., ibuprofen, diclofenac) (used as representative agents in separate nanoparticle-based delivery systems)	Corticosteroid (dexamethasone); polyphenolic anti-inflammatory agent (curcumin); non-steroidal anti-inflammatory drugs (NSAIDs)	Enable targeted delivery to inflamed tissues, enhance local drug accumulation, improve solubility and stability of poorly water-soluble anti-inflammatory agents, and reduce systemic toxicity associated with chronic anti-inflammatory therapy	Widely investigated in lipid, polymeric, and hybrid nanoparticle systems for inflammatory diseases, particularly rheumatoid arthritis and inflammatory bowel disease (IBD). Preclinical studies demonstrate improved tissue targeting, sustained release, and reduced systemic adverse effects compared to free drugs	[63,64,65,66,67]
**Neurological disorders**	Dopamine agonists; siRNA; neuroprotective agents (representative CNS therapeutic classes)	Small-molecule CNS drugs; gene therapy (RNA-based therapeutics); neuroprotective compounds	Facilitate crossing of the blood–brain barrier (BBB), enhance central nervous system (CNS) bioavailability, protect labile therapeutic agents from degradation, and improve targeted delivery to neuronal tissues	Rapidly expanding field of nanomedicine focused on neurological disorders such as Parkinson’s and Alzheimer’s diseases. Lipid–polymer hybrid nanoparticles and other engineered nanocarriers are being extensively investigated to overcome BBB limitations and improve CNS drug delivery efficiency. Most applications remain at preclinical and early translational stages	[68,69,70]

Another aspect that deserves greater attention is the role of biobanks in supporting the development of LPHNP-based therapeutics. Although biobanks are rarely discussed in the LPHNP literature, they may provide important opportunities to strengthen the translational relevance of preclinical research. Contemporary biobanks increasingly combine biological specimens with genomic, molecular and clinical information, creating valuable resources for biomarker discovery, patient stratification and therapeutic target validation. In addition, patient-derived samples and organoid biobanks can provide experimental models that more closely reflect human disease heterogeneity than conventional cell culture systems. Their integration into LPHNP research could facilitate the evaluation of nanoparticle performance in clinically relevant settings and contribute to the development of more personalized delivery strategies. Future studies should therefore explore stronger links between nanomedicine research and biobank infrastructures, particularly in the context of precision medicine and biomarker-guided therapeutic approaches [71,72,73].

### 7.4. Genomic Profiling: Tailoring Therapies Based on Tumor Biomarkers

Genomic and molecular profiling has become a cornerstone of modern oncology, enabling the implementation of precision medicine through the identification of actionable tumor biomarkers. By analyzing DNA, RNA, and protein alterations in tumor tissue, clinicians can detect genetic variants that guide therapeutic decision-making. This approach is facilitated by high-throughput technologies, particularly next-generation sequencing (NGS), which allows rapid and comprehensive assessment of cancer-related gene panels from clinical samples [74]. Consequently, molecular profiling is increasingly incorporated into routine diagnostic workflows at diagnosis and relapse to identify predictive and prognostic biomarkers [75].

Clinically actionable examples include the identification of EGFR mutations in non-small cell lung cancer, which predict response to EGFR tyrosine kinase inhibitors such as osimertinib, and BRAF V600E mutations in melanoma, which support the use of BRAF/MEK inhibitor combinations. Additional genomic biomarkers, including tumor mutational burden (TMB) and microsatellite instability (MSI), are used to predict responsiveness to immune checkpoint inhibitors. The expansion of such genotype–drug associations has enabled the development of basket trial designs, in which patients are enrolled based on shared molecular alterations rather than tumor histology [76,77]. However, a subset of tumors lacks clearly targetable driver mutations, necessitating alternative strategies such as clinical trial enrollment or combination therapies guided by resistance mechanisms.

The expertise of pharmacists is increasingly important in facilitating the translation of genomic data into clinically actionable treatment strategies. In multidisciplinary Molecular Tumor Boards, pharmacists collaborate with oncologists, pathologists, and geneticists to interpret genomic findings and support therapeutic selection [78]. Their expertise is essential in evaluating drug appropriateness, optimizing dosing strategies, assessing potential drug–gene interactions, and determining formulation feasibility, including the need for compounding in specific cases. Pharmacists also contribute to the management of resistance mechanisms, such as BCR-ABL mutations in chronic myeloid leukemia, where specific mutation profiles (e.g., T315I) guide the selection of second- or third-generation tyrosine kinase inhibitors [79,80].

Pharmacogenomic considerations further extend to supportive care. Variants affecting drug metabolism, such as DPYD variants associated with dihydropyrimidine dehydrogenase (DPD) deficiency and an increased risk of fluoropyrimidine toxicity, or CYP2D6 polymorphisms affecting opioid metabolism, may require dose adjustment or alternative therapy selection to ensure safety and efficacy [81]. These interventions highlight the importance of integrating pharmacogenomic data into both anticancer and supportive care regimens.

From a pharmaceutical compounding perspective, genomic profiling may necessitate individualized formulations, particularly in pediatric oncology or rare tumor subtypes. Tumor-agnostic therapies such as larotrectinib and entrectinib, approved for NTRK fusion-positive cancers, may require patient-specific dosing adjustments or liquid formulations for pediatric use [82,83,84]. In such cases, pharmacists ensure the preparation of stable and accurate compounded formulations to support access to targeted therapies across all patient populations. Overall, genomic profiling has substantially advanced personalized oncology by enabling treatment strategies tailored to individual tumor biology. This progress has improved clinical outcomes in multiple cancer types while increasing the complexity of therapeutic decision-making. Accordingly, pharmacists are becoming increasingly involved in the interpretation of molecular data, optimization of targeted therapies, and support of individualized pharmaceutical preparations. As genomic data becomes increasingly complex, integration with computational and artificial intelligence tools is expected to support clinical interpretation; however, expert pharmacological input remains essential for safe and effective implementation. Another aspect is the interpretation of pharmacogenomic data for supportive care. For example, if a patient has a genetic variant affecting drug metabolism, dihydropyrimidine dehydrogenase deficiency, which affects 5-FU metabolism, or a CYP2D6 poor metabolizer status that could influence codeine efficacy for pain, the pharmacist will adjust medication choices accordingly [79]. These interventions highlight the importance of integrating pharmacogenomic data into both anticancer and supportive care regimens.

Overall, genomic profiling has substantially advanced personalized oncology by enabling treatment strategies tailored to individual tumor biology. This progress has improved clinical outcomes in multiple cancer types while increasing the complexity of therapeutic decision-making.

### 7.5. Combination Therapies: Strategies to Enhance Efficacy and Overcome Therapeutic Resistance

Combination therapy, defined as the concurrent use of two or more therapeutic agents or modalities, is a fundamental strategy in oncology aimed at improving treatment efficacy and delaying the development of resistance. Cancer cells can adapt to single-agent therapies through activation of compensatory signaling pathways or acquisition of resistance mutations. The use of combination regimens targeting multiple pathways simultaneously reduces the likelihood of tumor escape and enhances therapeutic outcomes.

Historically, combination chemotherapy regimens such as ABVD for Hodgkin lymphoma or gemcitabine plus nab-paclitaxel for pancreatic cancer have demonstrated improved efficacy by leveraging drugs with complementary mechanisms of action [85]. More recently, combination approaches have expanded to include multimodal strategies, such as the integration of targeted therapies, immunotherapies, and hormone therapies, to exploit synergistic effects and improve clinical response.

Clinical evidence supports the effectiveness of these strategies. In metastatic melanoma, dual immune checkpoint blockade with ipilimumab and nivolumab has demonstrated superior response rates and survival compared with monotherapy [86]. In non-small cell lung cancer, the combination of chemotherapy with immunotherapy (e.g., carboplatin/pemetrexed plus pembrolizumab) has become a standard first-line treatment for many patients [87]. Similarly, targeted therapy combinations, such as BRAF and MEK inhibitors in melanoma, have been shown to delay resistance and improve outcomes [88]. Additional approaches, including combinations of targeted agents with anti-angiogenic therapies or the integration of radiotherapy with immunotherapy, are under investigation [89].

Despite these advances, combination therapies present significant challenges. The risk of overlapping or cumulative toxicities requires careful evaluation, and the optimization of dosing schedules remains complex, as both simultaneous and sequential administration strategies may be necessary depending on the therapeutic context. The safe implementation of combination regimens relies on pharmacists’ expertise in assessing potential drug–drug interactions, supporting dose optimization, and monitoring for adverse effects. They also contribute to the development of standardized treatment protocols and ensure the appropriate inclusion of supportive care measures, such as antiemetics, growth factors, and hydration strategies, to enhance treatment tolerability [90].

From a pharmaceutical compounding perspective, combination therapy may involve the preparation of multi-agent intravenous (IV) infusions. When drugs are chemically and physically compatible, pharmacists can compound multiple agents into a single infusion, reducing the number of administrations required. This approach requires strict adherence to validated compatibility and stability data to ensure product integrity and patient safety. In cases of incompatibility, agents must be administered separately, with pharmacists contributing to the design of administration schedules based on pharmacokinetic properties, interaction potential, and protocol requirements.

In outpatient settings, oral combination therapies are frequently used and may involve complex dosing schedules. Pharmacists contribute to treatment adherence through patient education, medication counseling, and the provision of adherence-support tools such as customized packaging or dosing calendars. These interventions are particularly important when regimens involve alternating schedules or cycle-based administration [90]. Advances in precision oncology have introduced the concept of genomically guided combination therapies, in which multiple targeted agents are selected based on the molecular profile of an individual tumor. Although still largely experimental, such approaches may involve the use of several agents targeting distinct oncogenic pathways [5]. Pharmacists are essential in evaluating the feasibility of these regimens, including the assessment of pharmacokinetic interactions, cumulative toxicities, and monitoring strategies.

Combination strategies have significantly improved outcomes in several malignancies. For example, concurrent chemoradiotherapy in locally advanced cervical cancer and the addition of anti-HER2 therapy to chemotherapy in HER2-positive breast cancer have markedly improved survival and cure rates [86,91]. Ongoing research continues to explore optimal therapeutic combinations, including multi-modality regimens integrating immunotherapy, targeted therapy, and cytotoxic agents.

Overall, combination therapies represent a critical strategy for enhancing therapeutic efficacy and overcoming resistance in oncology. Pharmacists are central to the optimization of these complex regimens, ensuring accurate preparation, safe administration, and effective patient monitoring, while also supporting adherence and patient education throughout the treatment continuum.

## 8. New Technological Approaches in Personalized Cancer Medicine

Advances in technology are continually opening new frontiers in how we design, compound, and deliver oncology treatments. The pharmacy profession, in particular, has seen a surge of innovative tools and methodologies that enhance personalized medicine. In this section, we highlight several key technological approaches: artificial intelligence, 3D printing of medications, and robotic automation that are transforming oncology pharmacy practice and the development of tailored cancer therapies.

### 8.1. AI-Based Algorithms and Big Data in Oncology

Artificial intelligence (AI) and machine learning algorithms are increasingly being integrated into oncology to support precision medicine across diagnostics, prognostics, and therapeutic decision-making. These systems can analyze large-scale and heterogeneous datasets, including electronic health records, genomic sequencing data, radiological imaging, histopathology slides, and real-time clinical monitoring data, to identify clinically relevant patterns that may not be readily apparent through conventional analysis. In precision oncology, machine learning models have been developed to predict treatment response by integrating tumor molecular profiles with clinical outcomes derived from large patient cohorts, thereby supporting individualized therapeutic selection, particularly in complex or rare clinical scenarios [92,93].

In drug development and optimization, AI-based approaches are used to accelerate compound screening, identify potential drug repurposing opportunities, and optimize combination therapies. Deep learning models can simulate drug–target interactions and predict pharmacodynamic and pharmacokinetic behavior, enabling the identification of novel therapeutic candidates and rational combination strategies. In addition, AI systems incorporating pharmacogenomic data and physiological parameters can support the development of individualized dosing regimens aimed at maximizing efficacy while minimizing toxicity [94,95].

In clinical practice, AI has also advanced diagnostic precision through radiomics and digital pathology. Imaging-based algorithms can extract quantitative features from CT and MRI scans to predict tumor phenotype and molecular characteristics, while pathology-based models can assess histomorphological and microenvironmental features associated with prognosis and treatment response [96]. Integrated AI platforms combining imaging and genomic data have demonstrated the ability to support treatment recommendations consistent with expert multidisciplinary tumor boards, highlighting their potential role as decision-support tools in oncology care [97]. Within pharmacy practice, AI applications are emerging in medication safety, compounding workflows, and clinical decision support. Automated systems can assist in verifying dose accuracy, detecting preparation errors, and ensuring correct labeling in sterile compounding environments. Natural language processing tools can analyze patient records and laboratory data to identify clinically relevant alerts, such as renal impairment requiring chemotherapy dose adjustment. These technologies enhance pharmacist-led medication safety processes, particularly in high-volume oncology settings [98].

AI is also being explored in treatment scheduling and chronotherapy, where dosing regimens are optimized according to biological rhythms and patient-specific response patterns. Machine learning models can evaluate toxicity profiles and tumor dynamics to propose individualized dosing intervals or cycle modifications, extending beyond conventional fixed-schedule chemotherapy protocols [99].

Despite these advances, AI should be regarded as a decision-support tool rather than a replacement for clinical expertise. Its clinical utility depends on data quality, transparency, and validation across diverse patient populations. Concerns related to algorithmic bias, data representativeness, and patient privacy remain critical considerations for safe and equitable implementation.

Overall, AI-based systems are expected to significantly enhance personalized oncology by integrating multidimensional clinical and molecular data into actionable insights. Their application spans drug discovery, diagnostic refinement, treatment selection, and pharmacy operations. Pharmacists, as integral members of the oncology care team, are increasingly positioned to leverage these technologies to improve medication safety, optimize therapy selection, and support individualized compounding and dispensing practices.

### 8.2. 3D-Printing and Personalized Drug Formulations

Three-dimensional (3D) printing, also known as additive manufacturing, is an emerging pharmaceutical technology with significant potential to transform personalized oncology therapy. It enables the on-demand production of solid dosage forms with precise control over dose, geometry, drug distribution, and release kinetics, thereby supporting highly individualized treatment strategies [100]. A major advantage of 3D printing is the ability to fabricate complex drug delivery systems, including multi-layered tablets and multi-compartment formulations. These structures allow for the incorporation of different drugs within a single dosage form, enabling immediate, sustained, or delayed release profiles tailored to patient-specific pharmacokinetic requirements [101]. This is particularly relevant in oncology, where patients often require concurrent medications for pain control, antiemesis, and supportive care.

3D printing also facilitates the development of polypills, which combine multiple active pharmaceutical ingredients into a single unit to simplify complex therapeutic regimens. This approach may improve adherence and reduce pill burden in cancer patients receiving polypharmacy for symptom management and comorbid conditions [102].

In pediatric oncology and special populations, 3D printing offers a solution to the long-standing challenge of dose inaccuracy and formulation limitations. Patient-specific doses can be manufactured as mini-tablets or rapidly dissolving formulations precisely adjusted to body weight or surface area. This avoids the need for manipulation of adult dosage forms and improves dosing accuracy and acceptability. The first FDA-approved 3D-printed medication, Spritam^®^ (levetiracetam), demonstrates the feasibility of this technology in clinical practice and provides a model for future oncology applications [103].

Beyond oral dosage forms, 3D printing is also being explored for localized drug delivery systems. Implantable scaffolds and drug-loaded patches have been developed to deliver chemotherapeutic agents directly to tumor sites, achieving sustained local release while minimizing systemic toxicity. Commonly investigated biodegradable polymers such as poly(lactic-co-glycolic acid) (PLGA) and polycaprolactone (PCL) are widely used due to their tunable degradation profiles and compatibility with drug incorporation. For example, implantable polymer-based systems have shown potential in preclinical models of solid tumors by maintaining high local drug concentrations and inhibiting tumor growth [104,105]. In this context, additive manufacturing techniques such as fused deposition modeling (FDM) and related extrusion-based approaches enable precise control over scaffold architecture and drug release kinetics.

Recent advances have further expanded the role of 3D printing from dose customization toward the development of truly precision-based pharmaceutical products. In oncology, patient-specific formulations can increasingly be designed using clinical, pharmacogenomic, and biomarker data, enabling the production of dosage forms tailored not only to individual dosing requirements but also to disease characteristics and therapeutic response profiles. This approach aligns with the broader transition toward precision oncology, where treatment strategies are progressively adapted to the molecular features of each patient and tumor [106,107].

Another emerging trend is the implementation of decentralized and on-demand manufacturing models within hospital pharmacies. Rather than relying exclusively on centralized industrial production, 3D printing technologies may enable healthcare institutions to manufacture personalized medicines at the point of care, reducing preparation times and improving treatment flexibility. Such a model could be particularly valuable in oncology, where dose adjustments, treatment interruptions, and individualized therapeutic regimens are common [107,108].

Recent research has also highlighted the importance of Quality-by-Design (QbD) principles and Quality Target Product Profiles (QTPPs) in the development of 3D-printed medicines. These frameworks facilitate the systematic optimization of formulation composition, printing parameters, and product performance, helping to ensure reproducibility, product quality, and regulatory compliance. In parallel, increasing attention is being directed toward the harmonization of regulatory requirements for 3D-printed pharmaceuticals, particularly regarding raw material characterization, process validation, quality assurance, and the distinction between industrial manufacturing and extemporaneous compounding practices [109,110].

Furthermore, the integration of digital technologies, including artificial intelligence and advanced computational modeling, is expected to further accelerate the adoption of 3D printing in personalized medicine. Predictive algorithms may support formulation design, optimize printing parameters, and forecast drug release behavior, thereby enabling more efficient development of patient-specific dosage forms. Collectively, these advances position 3D printing as a key enabling technology for the future of precision pharmaceutical care [111,112].

Despite its promise, clinical translation of 3D-printed pharmaceuticals requires rigorous quality control, including validation of dose uniformity, stability, and mechanical integrity. Pharmacists will be central to this process, ensuring the safe implementation of printed dosage forms within hospital and clinical pharmacy settings. Future pharmacy practice may incorporate digital formulation design and on-demand manufacturing, representing a paradigm shift from traditional compounding toward precision pharmaceutical engineering [113,114].

### 8.3. Robotic Compounding Systems and Automation

Ensuring safety and accuracy in the preparation of hazardous chemotherapy agents is a critical priority in oncology pharmacy. In recent years, robotic compounding systems have emerged as an innovative solution to enhance the precision, efficiency, and safety of cytotoxic drug preparation. These automated platforms are designed to perform reconstitution, transfer, and mixing of injectable medications with minimal human intervention. Systems such as APOTECAchemo, Cytocare, and KIRO Oncology are increasingly implemented in hospital and cancer center settings worldwide.

Robotic compounding offers several key advantages. First, these systems provide high levels of dosing accuracy and reproducibility, reducing variability associated with manual preparation. Studies comparing robotic and human compounding have demonstrated that robotic systems consistently prepare doses within narrow margins of error, achieving high compliance with pharmacopeial accuracy standards. Integrated quality control mechanisms, such as gravimetric verification and in some cases spectroscopic monitoring, further enhance product reliability [115].

Second, robotic systems significantly reduce occupational exposure to hazardous drugs. Traditional manual compounding carries inherent risks of contamination through spills, aerosolization, or accidental contact, even when protective equipment is used. In contrast, robotic systems operate within closed, controlled environments, often equipped with negative pressure and high-efficiency particulate air (HEPA) filtration. This minimizes direct handling of cytotoxic agents and has been associated with reduced surface and product contamination compared with manual preparation [115,116].

Third, automation improves workflow efficiency in high-volume oncology pharmacy settings. Robotic systems can prepare multiple individualized doses in advance, supporting batch preparation and enabling earlier clinical review and administration. However, reported time savings are variable, as some studies indicate that manual preparation may be faster for simpler tasks, highlighting the need for workflow optimization and task allocation between humans and machines [115,116].

Despite automation, pharmacist oversight remains essential. Pharmacists are responsible for clinical verification of prescriptions prior to robotic execution, validation of the final compounded product, and resolution of any discrepancies or system errors. Routine calibration, maintenance, and quality assurance procedures are also required to ensure consistent performance and safety.

Therefore, robotic systems function as adjuncts rather than replacements, supporting pharmacists in delivering complex therapies safely and efficiently.

Overall, robotic compounding systems represent a significant advancement in oncology pharmacy practice. When supported by appropriate calibration, preventive maintenance, quality assurance procedures, and pharmacist oversight, they enhance dosing accuracy, reduce occupational exposure to hazardous drugs, and improve workflow efficiency while maintaining high safety standards. As these technologies continue to evolve, further integration with artificial intelligence and digital health systems is expected to expand the role of automation in personalized chemotherapy preparation, while reinforcing pharmacists’ responsibility for clinical supervision, quality management, and the safe implementation of advanced compounding technologies [115,117].

## 9. Safety, Regulations, and Challenges in Compounded Oncology Medications

The compounding and handling of oncology medicines, particularly hazardous antineoplastic agents, are governed by strict safety standards and regulatory frameworks designed to protect patients, healthcare professionals, and the environment. This section outlines the principal regulatory guidelines for compounded oncology medications, discusses key challenges in maintaining safety and compliance in clinical practice, and emphasizes pharmacists’ contribution to the implementation of evidence-based standards.

Several international and national organizations have developed comprehensive guidelines for the safe handling of hazardous drugs (HDs), including chemotherapeutic agents. In the United States, USP <800> (Hazardous Drugs—Handling in Healthcare Settings) defines mandatory requirements for facility design, including externally vented containment systems, negative-pressure rooms, engineering controls, decontamination procedures, and personnel training, as well as the use of personal protective equipment (PPE) during all stages of handling cytotoxic agents [118]. Similarly, the European Society of Oncology Pharmacy (ESOP) [119] and the International Society of Oncology Pharmacy Practitioners (ISOPP) provide internationally recognized best-practice standards. The World Health Organization (WHO) also contributes global recommendations for the safe management of hazardous pharmaceuticals, particularly in resource-variable healthcare systems.

A major ongoing challenge in oncology compounding is minimizing occupational exposure to hazardous drugs. Chronic exposure, even at low levels, has been associated with adverse health outcomes, including dermatological effects, reproductive toxicity, and a potential increased risk of secondary malignancies among healthcare workers. As a result, regulatory frameworks emphasize the use of engineering controls, such as biological safety cabinets and closed-system transfer devices (CSTDs), in combination with administrative controls and PPE (e.g., double gloves). The safety measures include staff training, competency assessment, and enforcement of aseptic compounding practices.

Environmental contamination monitoring is another critical component of safety assurance. Many institutions routinely perform surface wipe sampling in compounding and administration areas to detect residual cytotoxic contamination. These assessments serve as quality control measures for cleaning procedures and workflow integrity. When contamination levels exceed acceptable thresholds, corrective actions are implemented, including retraining of staff, workflow redesign, and reinforcement of cleaning protocols [120].

## 10. Quality Assurance and Beyond-Use Dating

Unlike commercially manufactured pharmaceuticals, compounded oncology preparations often lack extensive, long-term stability data. As a result, pharmacists rely on published stability studies, pharmacopeial guidance, or institution-specific validation data to determine appropriate storage conditions and beyond-use dates (BUDs). Regulatory frameworks generally apply conservative BUDs to ensure both sterility and potency of compounded sterile preparations. For example, typical guidance may limit use of sterile, preservative-free preparations to 24 h at controlled room temperature or 3–7 days under refrigeration, unless validated stability data support extended dating.

Recent updates to USP <797> allow for longer BUDs under specific validated conditions, including sterility testing and risk-based environmental controls. However, many oncology pharmacies continue to adopt conservative limits due to the high-risk nature of cytotoxic therapies and the critical importance of maintaining sterility and chemical stability. This creates operational challenges, as compounded doses often need to be prepared close to the time of administration, requiring efficient workflow coordination and just-in-time preparation strategies.

Future advances in oncology compounding may increasingly rely on stability-indicating analytical methods to generate robust evidence supporting beyond-use dating. Techniques such as high-performance liquid chromatography (HPLC), particularly when validated as stability-indicating methods, can detect drug degradation products and quantify changes in active pharmaceutical ingredient concentration over time under defined storage conditions. When combined with sterility testing, physicochemical characterization, and risk-based quality assurance, these analytical approaches may enable the establishment of evidence-based beyond-use dates for selected compounded oncology preparations. Such strategies have the potential to reduce unnecessary drug wastage, improve workflow efficiency, and optimize the use of high-cost antineoplastic agents while maintaining patient safety and regulatory compliance.

In addition to stability considerations, the safe disposal of compounded hazardous drugs and related materials is a critical component of quality assurance. Regulatory frameworks such as U.S. Environmental Protection Agency (EPA) guidelines [121] and NIOSH recommendations require strict segregation and disposal of cytotoxic waste, including unused drugs, contaminated consumables (e.g., syringes, IV bags, gloves), and trace-contaminated materials. Healthcare facilities typically implement color-coded waste systems (e.g., yellow or purple containers) to ensure appropriate segregation of hazardous waste streams.

Pharmacists also contribute substantially to education and risk mitigation beyond the clinical setting. This includes training healthcare staff on safe handling procedures and counseling patients receiving oral chemotherapy on safe home disposal practices, particularly regarding body fluids and unused medication. These measures are essential to prevent secondary exposure in domestic environments.

Regulatory oversight of compounding practices varies across countries but consistently emphasizes pharmacist accountability and documentation. For example, Brazil’s Conselho Federal de Farmácia (CFF) Resolution No. 288/1996 defines professional responsibilities for handling antineoplastic agents [122], while Portugal’s Portaria no. 594/2004 establishes best practice standards for pharmaceutical compounding [123]. In the United States, the Drug Quality and Security Act strengthened regulatory control by distinguishing between traditional compounding pharmacies and FDA-registered outsourcing facilities (503B entities), which are permitted to produce larger batches of sterile preparations under stricter quality standards. As a result, many hospitals now utilize 503B facilities for standardized high-risk preparations while retaining patient-specific compounding within hospital pharmacy settings [124].

## 11. Challenges and Areas for Improvement

Despite existing guidelines, surveys show that compliance is not uniform across institutions. Some centers, particularly those with limited resources, struggle to implement all recommended safety controls. For instance, not all facilities use closed-system transfer devices (CSTDs) due to their cost, even though these systems have been shown to reduce surface contamination. Ensuring comprehensive and continuous training for all staff remains an ongoing challenge: new nurses and pharmacists must be properly oriented to safe handling procedures, and periodic refresher training is essential to maintain compliance. In addition, language barriers and variations in local practices can further hinder the uniform adoption of international standards.

As oncologic therapies continue to evolve, guidelines must also be updated accordingly. The increasing use of oral antineoplastic agents and immunotherapies, including monoclonal antibodies, expands the scope of safe handling requirements beyond conventional cytotoxic chemotherapy, requiring ongoing revision of institutional protocols.

Pharmacists often take the lead in advocating for safety measures within healthcare institutions, presenting to hospital administration the rationale for investment in appropriate infrastructure, including worker protection, regulatory compliance, and long-term institutional safety. They also contribute to research on safer handling practices, such as evaluating decontamination techniques for hazardous drug spills, for example, the use of sodium hypochlorite or other neutralizing agents, and assessing new materials for personal protective equipment (PPE).

Patient safety in the context of compounded sterile preparations also remains a critical concern. Although rare, serious contamination incidents have occurred, most notably the 2012 fungal meningitis outbreak linked to contaminated steroid injections prepared at a U.S. compounding facility, although this event was not related to oncology [125]. In oncology practice, compounding errors can have severe consequences; for example, historical cases of extreme dosing errors, including 1000-fold overdoses, have been reported. As a result, regulatory frameworks increasingly emphasize automation, standardized procedures, and independent verification systems, such as barcode scanning and robotic compounding, to minimize human error. Pharmacists and healthcare institutions are also encouraged to systematically report errors and near-misses, enabling shared learning and prevention of future incidents.

Overall, the safe compounding of oncology medications is governed by a comprehensive regulatory framework that requires strict and consistent implementation. Pharmacists are central to this process, developing and enforcing protocols aligned with regulatory standards, providing education and training to compounding personnel, and ensuring ongoing compliance monitoring. Ongoing challenges include ensuring consistent adherence to protective measures, keeping pace with evolving regulations, and maintaining product quality in high-pressure clinical environments. Nevertheless, through adherence to established standards and continuous improvement, such as the adoption of advanced compounding technologies and updated training programs, pharmacies worldwide strive to ensure that personalized cancer therapies are prepared as safely as possible. This approach allows patients to benefit from individualized treatment while safeguarding healthcare workers and preserving the quality and integrity of compounded medications.

## 12. Oncology Pharmacists in Compounding, Safety, and Multidisciplinary Cancer Care

Oncology pharmacists are central to the individualization of cancer treatment through the compounding, validation, and safe handling of antineoplastic drugs. These highly trained professionals bridge the physician’s therapeutic plan and the safe delivery of customized therapy to the patient. Key responsibilities include selection of appropriate formulations, precise preparation of compounded doses, quality control, dispensing, and patient counseling [126].

In essence, the pharmacist ensures that each oncology medication is delivered in the correct pharmaceutical form, strength, and combination as intended by the multidisciplinary oncology team, while simultaneously safeguarding against errors and contamination. In accordance with legal and professional standards, oncology pharmacists act as the final checkpoint for treatment safety. This includes reviewing chemotherapy prescriptions for dosing accuracy based on patient-specific factors such as body surface area and organ function, assessing potential drug–drug interactions or therapeutic duplications, and ensuring alignment with established clinical protocols and guidelines.

In Portugal, as in many other countries, national legislation further reinforces these responsibilities. For example, Decree-Law No. 95/2004 mandates pharmacist verification of compounded medications, including dose accuracy, compatibility of active substances, and absence of contraindicated interactions. When discrepancies are identified, pharmacists collaborate closely with oncologists and nursing staff to resolve and validate prescriptions before compounding proceeds. Evidence demonstrates that the integration of oncology pharmacists into the medication ordering process significantly reduces chemotherapy errors and improves patient safety outcomes [90]. Pharmacist-led interventions have also been associated with improved medication safety and enhanced supportive care monitoring [12]. Consequently, oncology pharmacists are increasingly recognized as essential members of multidisciplinary cancer care teams, often participating in tumor boards and clinical ward rounds [127].

Compounded medications generally fall into two main categories. Magistral preparations are patient-specific formulations prepared according to a physician’s prescription, such as chemotherapy doses adjusted to body surface area or organ function. In contrast, officinal preparations are pharmacy-prepared formulations produced in advance according to pharmacopeial standards and stored for use [128]. Pharmacists must be proficient in both approaches, frequently adapting or developing formulations on demand.

Modern oncology practice frequently requires individualized solutions. These include preservative-free formulations for patients with excipient allergies, liquid preparations for frail patients or those unable to swallow solid dosage forms, and combination infusions designed to reduce fluid burden. In all cases, pharmacists assess compatibility and stability, ensuring therapeutic efficacy while preventing degradation or harmful interactions. Given the narrow therapeutic index of many antineoplastic agents, meticulous verification of calculations and measurements is essential. Final compounded products undergo rigorous quality checks, including volume accuracy, labeling, and visual inspection, often supported by independent double-checking systems [129].

Chemotherapy compounding, particularly for hazardous drugs, follows highly controlled protocols in specialized cleanroom facilities equipped with biological safety cabinets or isolators. Standards such as USP <800> and USP <797> define requirements for environmental control, including negative-pressure rooms, HEPA filtration, closed-system transfer devices, and appropriate personal protective equipment [118,130]. Pharmacists are responsible for ensuring compliance and often develop institution-specific standard operating procedures, while also training pharmacy technicians involved in compounding.

Technological safeguards further enhance accuracy and safety. Barcode verification systems and gravimetric analysis are widely used to confirm ingredient identity and dosing precision. Quality assurance may also include sterility testing and stability monitoring, particularly for preparations intended for delayed use or extended storage [131]. Pharmacists also ensure proper labeling, including drug identification, concentration, beyond-use dates, storage conditions, and handling instructions.

A major driver of compounding is the need for individualized therapy. Pediatric oncology patients often require dose adjustments or liquid formulations due to an inability to swallow tablets, while patients with dysphagia or enteral feeding tubes similarly depend on non-solid formulations. Compounding also ensures continuity of care when commercial products are discontinued. Additionally, when chemically compatible, multiple agents may be combined into a single dosage form to simplify regimens and improve adherence, particularly in patients receiving polypharmacy [127].

Oncology pharmacists’ responsibilities extend beyond technical preparation into education and clinical collaboration. They provide counseling to patients and caregivers on medication use, adverse effect management, and safe handling of hazardous drugs at home. Within the oncology team, they educate clinicians on formulation constraints and stability considerations, such as advising when dose modifications are not feasible due to physicochemical limitations.

Pharmacists also contribute to the development and optimization of treatment protocols, ensuring regimens are both evidence-based and practically implementable in a compounding environment. In many cancer centers, they actively participate in multidisciplinary meetings, reviewing complex cases and contributing to therapeutic optimization. Their involvement has been associated with improved patient satisfaction, better adherence, enhanced monitoring of toxicity (including laboratory follow-up), and reduced healthcare costs through optimized dosing and decreased waste [12].

As personalized medicine advances, oncology pharmacists are increasingly involved in molecular tumor boards, where genomic data are interpreted to identify targeted therapies for rare mutations.

In such contexts, pharmacists help ensure access to appropriate treatments, including through compounding or special procurement pathways when commercial formulations are unavailable.

Overall, the contribution of oncology pharmacists to compounded medications is indispensable. They ensure that personalized cancer therapies are translated into safe, effective, and practically deliverable treatments. They uphold stringent standards to protect patients and healthcare workers from hazardous drug exposure, while also advocating for patient-centered care through innovative pharmaceutical solutions. Working closely with physicians and nurses, pharmacists ensure safe implementation of treatment plans through prescription verification, individualized compounding, and patient education. Through this collaborative approach, therapy can be continuously tailored to patient-specific factors and adjusted in real time based on clinical response and tolerability [132].

## 13. Conclusions and Future Perspectives

Cancer care is undergoing a profound paradigm shift toward highly personalized therapy, and compounded oncology medications will remain a cornerstone of this evolution. This review has highlighted how advances in the understanding of cancer biology and molecular drivers have enabled the development of innovative therapeutic strategies, including targeted therapies, immunotherapies, and advanced drug delivery systems. Within this context, the expertise of pharmacists in compounding and medication management is essential to ensure that these innovations are translated into safe, effective, and patient-specific treatments.

Several key trends are expected to shape the future of oncology pharmacy. The continued integration of emerging therapies into clinical practice will require pharmacists to expand their competencies, particularly in relation to next-generation immunotherapies, oncolytic virotherapy, and personalized cancer vaccines. As gene and cell therapies become more widely adopted, pharmacists will increasingly engage in complex logistical processes, including cell handling, cryopreservation, and chain-of-custody management for these advanced “living medicines.” In parallel, patient education responsibilities are expected to expand in response to the complexity of these therapies, extending pharmacy practice beyond traditional compounding into the broader domain of advanced therapy medicinal products.

Technological innovation will play a transformative role in oncology pharmacy practice. The integration of robotics, artificial intelligence, and three-dimensional printing is expected to expand significantly within pharmacy workflows. Future models of care may enable the preparation of individualized treatment regimens, including anticancer agents, supportive therapies, and nutritional components, through automated systems informed by real-time clinical and laboratory data. These developments will require robust pharmacy informatics infrastructure and close interdisciplinary collaboration to validate and implement AI-driven dosing and automated compounding systems. Despite their considerable potential, the widespread implementation of these technologies in hospital pharmacy practice faces significant practical challenges. The adoption of robotic compounding systems, artificial intelligence platforms, and three-dimensional printing requires substantial capital investment, ongoing maintenance costs, and dedicated technical support, which may limit accessibility, particularly in resource-constrained healthcare settings. In addition, seamless interoperability between these technologies and existing electronic health records, computerized physician order entry, and pharmacy information systems remains a major challenge for ensuring efficient and secure data exchange. Regulatory frameworks have not yet fully evolved to address the routine clinical use of emerging technologies, particularly with respect to the quality assurance and governance of three-dimensional-printed medicines prepared within hospital pharmacies. Furthermore, successful implementation depends on specialized education and continuous training of pharmacists and technical staff to ensure the safe operation, validation, and oversight of increasingly automated compounding processes. Addressing these challenges will be essential to translate technological innovation into sustainable improvements in personalized oncology care [133]. Such technologies have the potential to enhance precision, reduce errors, and allow pharmacists to focus more on clinical decision-making and patient-centered care.

Future developments in pharmaceutical 3D printing are expected to rely increasingly on structured formulation and process development frameworks, particularly Quality-by-Design (QbD) approaches and Quality Target Product Profiles (QTPPs). These strategies will be essential to ensure the systematic design, optimization, and reproducibility of personalized dosage forms, especially as production moves closer to decentralized and hospital-based manufacturing settings. In parallel, the establishment of clearer and more harmonized regulatory pathways will be critical to support the clinical translation of 3D-printed medicines, particularly with regard to raw material qualification, process validation, and quality assurance requirements. Together, these developments are likely to strengthen the robustness, scalability, and clinical acceptance of additive manufacturing technologies, facilitating their integration into routine precision pharmaceutical care.

Advances in safety and regulatory compliance must evolve alongside these technological developments. Future innovations may include real-time exposure monitoring systems for hazardous drugs and AI-assisted tools to ensure adherence to cleanroom protocols and aseptic techniques. At the same time, regulatory frameworks will need to adapt to accommodate emerging therapeutic classes, including complex modalities such as radiopharmaceutical–antibody conjugates and other advanced therapies. Greater harmonization of regulatory standards and clearer policy guidance will be essential to ensure the safe, consistent, and equitable implementation of compounded and personalized treatments across healthcare systems. Pharmacists are central to the interpretation and operationalization of these evolving requirements in clinical practice.

Equally important is their expanding function as educators and patient advocates. As oncology therapies become increasingly individualized and complex, effective patient education is essential, particularly for oral anticancer agents administered in home settings. Pharmacists support patients and caregivers in medication adherence, adverse effect management, and safe handling practices, providing a crucial human interface within an increasingly technology-driven healthcare environment. While artificial intelligence may support treatment design, pharmacists remain essential in contextualizing therapy according to patient preferences, lifestyle, and clinical needs, thereby ensuring truly patient-centered care.

From a research and development perspective, pharmacists and pharmaceutical scientists will continue to contribute to advances in compounded formulations and innovative drug delivery systems. Future research will likely focus on the stability of complex admixtures, optimization of individualized dosing strategies, and the development of novel compounding technologies, including microfluidic approaches for nanomedicine. Collaboration between academia, clinical practice, and industry is expected to intensify, particularly in the development of personalized dosage forms and advanced therapeutic systems.

In conclusion, compounded medications remain an essential pillar of personalized oncology care, enabling a level of therapeutic individualization that cannot be achieved through conventional manufactured dosage forms alone. Within this framework, pharmacists are involved not only in preparation but also in the optimization, safety assurance, and clinical integration of individualized therapies. As oncology continues to evolve, pharmacists will increasingly operate at the interface of science, technology, and patient care, coordinating complex and highly individualized treatment strategies.

The ongoing challenge will be to balance innovation with safety and personalization with consistency and quality. With their expertise in pharmacotherapy, pharmacokinetics, and pharmaceutics, combined with a strong patient-centered approach, pharmacists are uniquely positioned to meet this challenge. Their collaboration within multidisciplinary oncology teams will remain fundamental in translating scientific advances into improved survival outcomes and quality of life, ultimately contributing to a more precise, effective, and humane model of cancer care.

## Figures and Tables

**Table 1:** Main Cancer Risk Factors.

Intrinsic (Unmodifiable)	Non-Intrinsic Exogenous (Partially Modifiable)	Non-Intrinsic Endogenous (Modifiable)
Random DNA replication errors	Hormonal imbalances	Chemical carcinogens (e.g., asbestos)
Genetic susceptibility	Chronic inflammation	Viral infections (e.g., HPV, HBV)
Inherited DNA repair defects	Growth factor dysregulation	Smoking, Alcohol consumption, Poor diet, and physical inactivity

## Data Availability

No new data were created or analyzed in this study. Data sharing is not applicable to this article.

## Abbreviations

AI	Artificial Intelligence
ALK	Anaplastic Lymphoma Kinase
ASR	Age-Standardized Rate
BCR-ABL	Breakpoint Cluster Region–Abelson Murine Leukaemia Viral Oncogene Homologue
BiTEs	Bispecific T-cell Engagers
BRAF	v-Raf Murine Sarcoma Viral Oncogene Homologue B
BRCA1/2	Breast Cancer Gene 1/Breast Cancer Gene 2
CAR-T	Chimeric Antigen Receptor T cells
CD	Cluster of Differentiation
CRISPR	Clustered Regularly Interspaced Short Palindromic Repeats
CTLA-4	Cytotoxic T-Lymphocyte–Associated Protein 4
DNA	Deoxyribonucleic Acid
DPYD	Dihydropyrimidine Dehydrogenase
EGFR	Epidermal Growth Factor Receptor
EPA	Environmental Protection Agency
FDA	Food and Drug Administration
HBV	Hepatitis B Virus
HER2	Human Epidermal Growth Factor Receptor 2
HPV	Human Papillomavirus
IARC	International Agency for Research on Cancer
ICIs	Immune Checkpoint Inhibitors
INFARMED	Autoridade Nacional do Medicamento e Produtos de Saúde, I.P.
irAEs	Immune-related Adverse Events
ISOPP	International Society of Oncology Pharmacy Practitioners
KRAS	Kirsten Rat Sarcoma Viral Oncogene Homologue
LPHNPs	Lipid–Polymer Hybrid Nanoparticles
mAbs	Monoclonal Antibodies
MEK	Mitogen-Activated Protein Kinase
MSI	Microsatellite Instability
NIOSH	National Institute for Occupational Safety and Health
NRAS	Neuroblastoma RAS Viral Oncogene Homologue
NTRK	Neurotrophic Tyrosine Receptor Kinase
PARP	Poly (ADP-ribose) Polymerase
PD-1	Programmed Cell Death Protein 1
PD-L1	Programmed Death-Ligand 1
USP	United States Pharmacopoeia
VEGF	Vascular Endothelial Growth Factor
VEGFR	Vascular Endothelial Growth Factor Receptor
WHO	World Health Organisation
3D	Three-Dimensional
